# Application of a computer-controlled flow analyser to the determination of glucose and to the study of β -galactosidase activity

**DOI:** 10.1155/S1463924692000178

**Published:** 1992

**Authors:** David J. Malcolme-Lawes, Koon Hung Wong, Brian V. Smith

**Affiliations:** 1 Centre for Research in Analytical Chemistry and Instrumentation King's College London Strand London WC2R 2LS UK; 2 Department of Chemistry King’s College London Strand London UK

## Abstract

Glucose was determined using an automated flow analyser and a commercial reagent preparation based on the glucose oxidaseperoxidase reaction. The coupling system for the hydrogen peroxide liberated consisted of 4-aminophenazone and 2,4-dichlorophenol. The analyser demonstrated linearity of>0.9997 for five consecutive sets of standards. The average RSD for individual standards is 0.76%, and a sample throughput rate of >80 h^-1^ was achieved.

The study of β-galactosidase, using a novel substrate, was carried out in a simple single-line manifold. The enzyme-substrate reaction mixture was injected into a pH 10 buffer carrier to stop the reaction, and at the same time, propel the reaction zone to the flow cell. K_m_ and V_max_ values were calculated.


Journal of Automatic Chemistry, Vol. 14, No. 3 (May–June 1992), pp. 73–78

### Application of a computer-controlled flow analyser to the determination of glucose and to the study of β-galactosidase activity

**David J. Malcolme-Lawes, Koon Hung Wong**
*Centre for Research in Analytical Chemistry and Instrumentation, King's College London, Strand, London WC2R 2LS*

**and Brian V. Smith**
*Department of Chemistry, King's College London, Strand, London, UK*

*Glucose was determined using an automated flow analyser and a commercial reagent preparation based on the glucose oxidase–peroxidase reaction. The coupling system for the hydrogen peroxide liberated consisted of 4-aminophenazone and 2,4-dichlorophenol. The analyser demonstrated linearity of >0·9997 for five consecutive sets of standards. The average RSD for individual standards is 0·76%, and a sample throughput rate of >80 $h^{-1}$ was achieved.*

*The study of β-galactosidase, using a novel substrate, was carried out in a simple single-line manifold. The enzyme-substrate reaction mixture was injected into a pH 10 buffer carrier to stop the reaction, and, at the same time, propel the reaction zone to the flow cell. $K_m$ and $V_{max}$ values were calculated.*

### Introduction

Glucose exists in two anomeric forms in nature, α-D-glucose and β-D-glucose, in the ratio of, roughly, one to two. Its determination is widely performed in the food industry and in clinical chemical laboratories: the diagnosis of diabetes is an important example of the latter [1]. Some of the methods rely on the reducing properties of the sugar, but the majority are based on enzymatic assays. Commonly used enzymes include glucose dehydrogenase (GDH), hexokinase (HK) and glucose oxidase (GOD) [2]. GDH is widely encountered, but it relies on the production of NADH which is monitored at 340 nm and is therefore unsuitable as a basis for a colorimetric reaction. Hexokinase, on the other hand, is not glucose-specific and would react with other hexoses. The specificity of a particular enzyme does not depend only on the biochemistry, but also on its source and the method of its commercial preparation. Assays using GOD are well documented and well-adapted to automatic analysers [3]. In this assay, β-D-glucose is oxidized to D-glucono-δ-lactone, which is subsequently converted to gluconic acid:

$$\beta\text{-D-glucose} + O_2 + H_2O + GOD \rightarrow \text{gluconic acid} + H_2O_2.$$

α-D-glucose oxidizes about 150 times slower than the beta-form, making this assay particularly specific. Species of interest to the analyst in the above reaction are oxygen and hydrogen peroxide. The rate of disappearance of oxygen and/or the formation of hydrogen peroxide could be measured manometrically, or by electrochemical detection. Colorimetric methods involve the reaction of hydrogen peroxide with one or more coupling reagents via peroxidase (POD) to produce a chromophore:

$$H_2O_2 + DH_2 + POD \rightarrow 2\ H_2O + D$$

where $DH_2$ is an oxygen acceptor (hydrogen donor), and D, the oxidized acceptor, is a chromophore. Examples of oxygen acceptors include o-dianisidine, o-tolidine, 2,2′-azino-bis-(3-ethylbenzthiazoline)-6-sulphonate (ABTS) and a combined phenol/4-aminophenazone system. o-Dianisidine and o-tolidine were originally used and formed the basis of 'test-strip' for glucose [3]; although these have been superseded owing to their carcinogenic properties. ABTS, although offering higher sensitivities, is relatively expensive. A simple assay developed by Barham and Trinder [4], based on a sulphonated 2,4-dichlorophenol/4-aminophenazone system offers four times the sensitivity over the basic phenol system.

Ruzicka and Hansen [5] adapted the GDH assay to flow-injection (FI) determination of glucose in blood serum in 1977. Many assays based on spectrophotometric detection have been adapted since then. Although Ruzicka and Hansen developed a glucose assay using soluble enzyme, the majority of later FI-practitioners have favoured the use of immobilized enzymes. Flow-injection workers have argued on the merits and disadvantages of using soluble enzymes and immobilizing them [6–8]. The overall conclusion appears to be that, for relatively inexpensive and robust enzymes, it is perfectly feasible to use them in solution, whereas immobilization may be a better option if the enzymes are delicate and/or expensive. Mottola [9] used GOD as one of the examples in comparing the two types of enzyme manipulation techniques. In solution, GOD retained over 50% of its original activity after more than 20 days. Mottola also found that, by using the enzyme in solution, a superior sample throughput could be achieved. For a low cost and robust enzyme such as GOD, he concluded that the time and effort consumed in immobilizing it could not be justified. Other workers have exploited the ability of enzymes to regenerate; Roehrig *et al.* [10] determined glucose in a closed-loop system by recycling and regenerating GDH. Despite what has been said, immobilized GOD reactors are dominant in the determination of glucose. Gorton and Ogren [11] used two enzyme reactors: firstly, the sample was dialysed before it was oxidized in the GOD reactor, and the oxidized stream then merged with a combined coupling reagent before it entered the POD reactor. Worsfold *et al.*

0142–0453/92 $3.00 © 1992 Taylor & Francis Ltd.

[12] chose to merge dialysed samples with the coupling reagent first before oxidation in the GOD column, POD was then introduced downstream to develop the reaction. An indirect determination of glucose was performed by Olsson *et al.* [13] who determined sucrose in the presence of glucose by using a 'glucose eliminating reactor'. In essence, this was a combined GOD-POD-mutarotase reactor, which took into account both anomers of glucose and also the hydrogen peroxide produced in the oxidation.

In enzymology, β-D-glucose can conveniently be considered as the substrate component of an enzyme-substrate reaction. The enzyme of interest in the second part of this study is β-galactosidase (β-D-galactoside galactohydrolase, EC 3.2.1.23). It is an important enzyme in the control of gene expression [14]. β-D-Galactosidase is an inducible enzyme which catalyses the hydrolysis of lactose to β-D-galactose and D-glucose [3]. Lactose is both the inducer and the substrate in the reaction. Sometimes, it is possible that an inducer is not a substrate, isopropylthiogalactoside (IPTG) is such a substance. In the study of β-galactosidase properties [4], o-nitrophenol-β-galactosidase (ONPG) is the most widely used substrate because it instantly ionises in alkaline solutions yielding a chromophoric product. A comprehensive review given by Wallenfels and Weil [15] suggests that maximal β-galactosidase activity occurs at between pH 7·2 and 7·4. $Na^+$ is said to activate the enzyme and although the activating effect of divalent ions is less clear cut, $Mg^{2+}$ ions are used in small amount in most assays. However, maximal activity for cleavage of ONPG shifts to pH 6·8 in the presence of $Mg^{++}$. The presence of certain alcohols also enhances the cleavage of ONPG by the enzyme by increasing the rate of one of the rate determining steps in the reaction. VLMgal, a novel substrate for β-galactosidase, in its cleavage by the enzyme should also be dependent upon some of the above parameters.

The applications of FIA to study the kinetics of enzymatic reactions have not been explored as much as might have been expected. Methods involving enzyme assays are frequently used in the determination of enzyme levels. These levels serve as an indicator of disease states, malfunctions of internal organs and bacterial infection, to name but three.

Application of FIA is thus seen as a valuable and practicable alternative to the conventional methods which depend on incubation. The availability of a new generation of substrates [16] makes this application an attractive one in the search for new applications. The released chromogens, as has been shown [17], are superior to those existing hitherto, and the high absorbance and stable colours which show maxima well removed from any biological background are additional advantages.

Olsen *et al.* [18] were quick to realize the potential of exploiting the gradient along the flow-injection profile, and, in effect, performed electronic dilution on the sample zone. By using an electronic timer, they were able to control the action of the pump accurately and measured the activity of lactose dehydrogenase by a stopped-flow technique. Riley *et al.* [19] also used the stopped-flow technique to study the kinetics of $\alpha_2$ macroglobulin. Kroner and Kula [20] determined the activity of alkaline protease by continuously pumping the enzyme and introduced the substrate-stream into it in a pulse-wise manner. Holm [21,22] evaluated enzyme activities in a more conventional manner by injecting enzyme standards into reagents and buffer streams. Direct injection of substrate-enzyme reaction mixtures are also common. Yoza *et al.* [23,24] measured the activity of inorganic pyrophosphatase, and Heller [25] with kidney aminoacyclase, by this means and observed the increase in the kinetic profiles as the reaction mixtures are pumped into the flow-cell. Only a handful of enzymes have been studied and there has been no publication devoted specifically to β-galactosidase.

### Experimental

The apparatus has remained virtually unchanged from that described in previous papers [26,27]. For the study of β-galactosidase an external water-bath was used. All the 'inorganic' reagents were of analytical grade, unless otherwise stated. Deionized water was filtered through 0·45 μm Durapore filter and de-gassed before use.

### *Materials*

Merckotest$^R$ kit for Glucose (3393), Merck.

β-Glactosidase (G-2513) from *E. coli*, Grade X, Sigma.

di-Sodium hydrogen orthophosphate, $Na_2HPO_4.12H_2O$, BDH.

Sodium dihydrogen orthophosphate, $NaH_2PO_4.2H_2O$, Fisons.

Magnesium chloride, $MgCl_2.6H_2O$, BDH.

Sodium hydrogen carbonate, $NaHCO_3$, SLR, Fisons.

Anhydrous sodium carbonate, $Na_2CO_3$, Fisons.

VLMgal was provided by BVS.

### *Determination of glucose*

The chemistry of the Merckotest kit was based on the coupling reaction suggested by Barham and Trinder [4]. Hydrogen peroxide liberated in the peroxidase reaction reacts with 4-aminophenazone and 2,4-dichlorophenol to form antipyrylchloroquinone imine. The reagent kit comprised a combined 0·1 M phosphate-tris buffer at pH 8·0, 6 kU/l GOD, 2·2 kU/l POD, 0·25 mM 4-aminophenol and 0·3 mM 2,4-dichlorophenol. The working reagent was prepared by adding deionized water (100 ml) to the reagent concentrate. Glucose standards were diluted from the 1000 mg/dl solution provided. Standards containing 40, 60, 100, 120 and 140 mg/dl of glucose were prepared covering the fasting blood glucose level of 60–100 mg % (mg glucose/100 ml blood)—levels of 120 mg % or above are indicative of diabetes. Both the reagent and the glucose standard solutions were kept in a thermostatted water-bath at 37 °C before use. 100 mg/dl glucose standard (0·1 ml) was added to the reagent (2·0 ml) in a cuvette. After mixing and incubating for 3 min the reaction mixture was scanned from 400 to 600 nm in a spectrophotometer. For the flow-injection calibration of glucose, water was found to be a suitable carrier with no problems derived from refractive index effects. The sensitivity of the assay was not considered important for such high levels of determinand and thus the calibration was optimized for linearity, sample throughput and reproducibility.

*Study of β-galactosidase*

The aim of this exercise was to assess the performance of a novel substrate, VLMgal, in these protocols, and to investigate the effects of temperature, divalent ion concentration and pH on the kinetics of the β-galactosidase cleavage of the substrate. Three protocols were used, each of them referring to the temperature in which the reaction was performed, the presence or absence of magnesium ions together with the pH of the buffer in which the enzyme solutions were prepared:

(A) 20 °C, 3 mM $Mg^{++}$, pH 6·8.
(B) 37 °C, no $Mg^{++}$, pH 6·8.
(C) 37 °C, 3 mM $Mg^{++}$, pH 8·0.

As the study of β-galactosidase lasted for several weeks, one underlying assumption made was that both the enzyme and the substrate had been little changed during the course of the study. To ensure that this was the case, the enzyme solution was stored at between 0 and 2 °C until it was required. A hydrolysis test conducted on VLMgal showed that it was stable at these pHs.

0·1 M phosphate buffer was prepared by mixing 0·1 M di-sodium hydrogen orthophosphate and 0·1 M sodium dihydrogen orthophosphate. The proportion of the orthophosphates was adjusted to give buffer solutions of pH 6·8 and pH 8·0. Where it was required, magnesium chloride (0·6099 g) was dissolved in phosphate buffer (1000 ml) to give a final solution 3 mM in magnesium ions. The stock enzyme solution had an initial activity of 285 U/mg protein (using ONPG as substrate). Upon dissolution and dilution 50 000 times, a working enzyme solution with 5·7 mU/mg activity was obtained. Standard solutions containing 0·05, 0·10, 0·15, 0·20, 0·25, 0·30, 0·40 and 0·50 mM of VLMgal were made by diluting a 1·0 mM working solution of the substrate with a concentration of 0·02907 g/50 ml. A 0·3 M carbonate buffer for stopping the enzyme reaction was prepared by dissolving sodium hydrogen carbonate (25·203 g) and sodium carbonate (31·677 g) in water (1000 ml). The carbonate buffer had a pH of 10. A preliminary experiment was conducted to estimate the $\lambda_{max}$ for protocols 'A' and 'B'. 0·5 ml of 5·7 mU/mg enzyme solution was mixed with 0·5 ml of 1 mM VLMgal. An incubation period of 4 min was allowed before 1 ml of carbonate buffer was added. A spectrum was taken between 500 and 600 nm. The procedure was carried out at room temperature for both protocol 'A' and 'B'. It was found that the two protocols gave almost identical peak shapes, with similarly broad absorption with $\lambda_{max}$'s at ~540 nm.

The control program for the flow analyser was modified slightly to facilitate accurate measurements of the time of injection and the occurrence of the peaks, i.e. a kinetic profile. A set of five standard VLMgal solutions were chosen for each protocol and all the solutions were kept in a thermostatted water-bath, except in the case of protocol 'A' whereby all the solutions were kept at room temperature. 2 ml of substrate was pipetted into a 10 $cm^3$ beaker, 2 ml of enzyme was added and a stop-watch was instantly started. An aliquot of the reaction mixture was selectively injected into the carbonate buffer carrier at regular intervals. The chromophoric product was monitored at 540 nm and a kinetic profile was collected for each substrate standard and for each protocol.

*Table 1. Flow conditions for glucose determination.*

| | |
|---|---|
| $\lambda_{max}$ | 510 nm |
| $t_{dil}$ | 1 s |
| $t_{fill}$ | 2·5 s |
| $t_{reac}$ | 8·0 s |
| *Coil temperature* | 38 °C |

*Table 2. The calibration characteristics for five sets of standards.*

| | | |
|---|---|---|
| *Correlation coefficient* | 0·9997 ± 0·0004 | |
| *Intercept* | 0·0291 ± 0·0079 | AU |
| *Sensitivity* | 0·4289 ± 0·0258 | AU/mg/ml |

*Table 3. Reproducibility of standard injection.*

| [Glucose] | Peak height | RSD |
|---|---|---|
| mg/dl | Mean AU | % |
| 400 | 0·1987 | 0·67 |
| 600 | 0·2697 | 0·60 |
| 1000 | 0·4184 | 1·21 |
| 1200 | 0·4987 | 0·54 |
| 1400 | 0·5225 | 0·76 |
| | Mean RSD = 0·76% | |

### Results

*Determination of glucose*

The optimized conditions and flow analyser parameters for the determination of glucose are given in table 1. The terminology is consistent with a previous paper [27].

Five sets of standard glucose solutions were injected under the conditions given above and their calibration characteristics are shown in table 2.

In order to estimate the reproducibility of the sample injection, each glucose standard was injection in 10 replicates consecutively and the results are given in table 3.

*Study of VLMgal as a substrate for β-galactosidase*

The results obtained from the flow injection studies of the VLMgal–β-galactosidase are presented in figures 1–3. There is almost a rectilinear relationship in all the kinetic

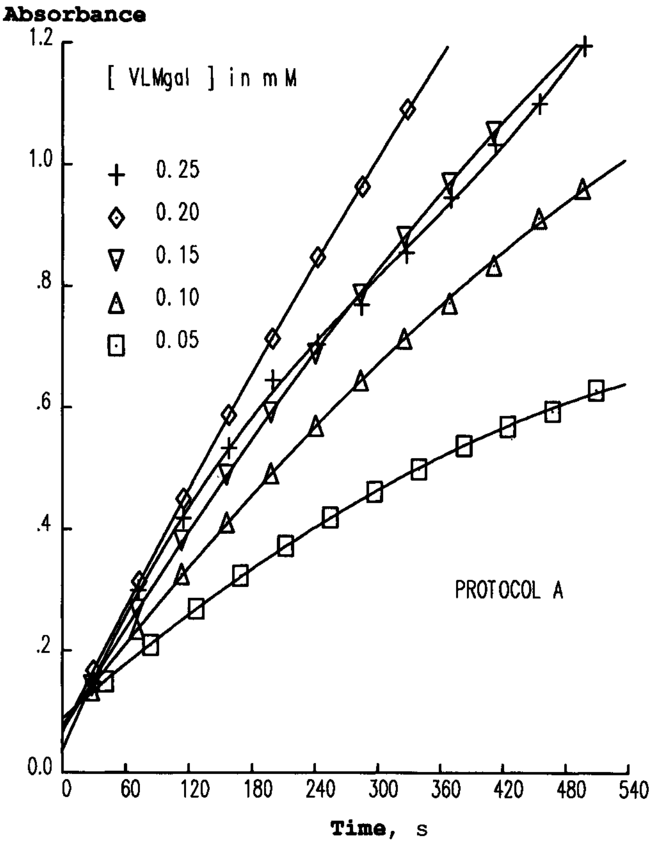

*Figure 1. Flow injection peak heights obtained for VLMgal-β-galactosidase using Protocol A.*

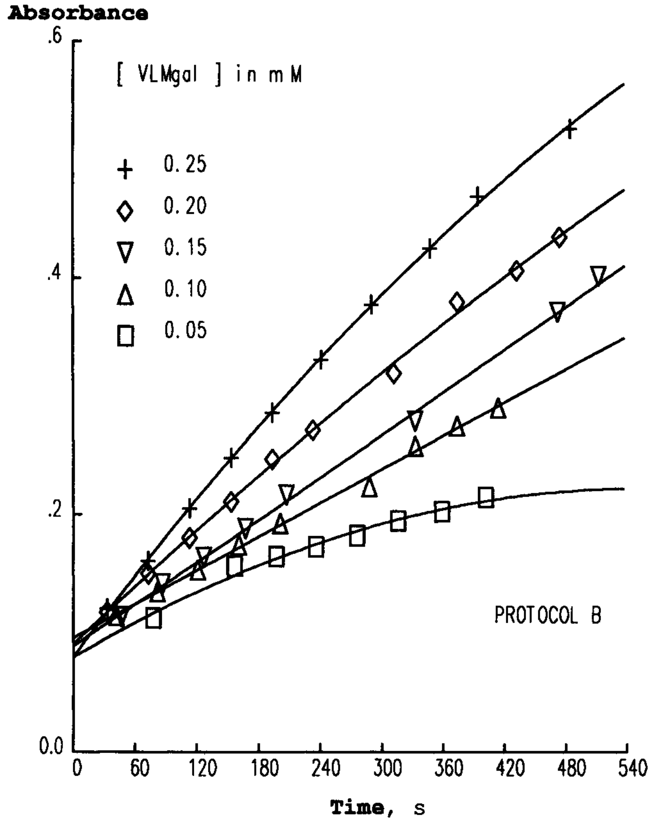

*Figure 2. Flow injection peak heights obtained for VLMgal-β-galactosidase using Protocol B.*

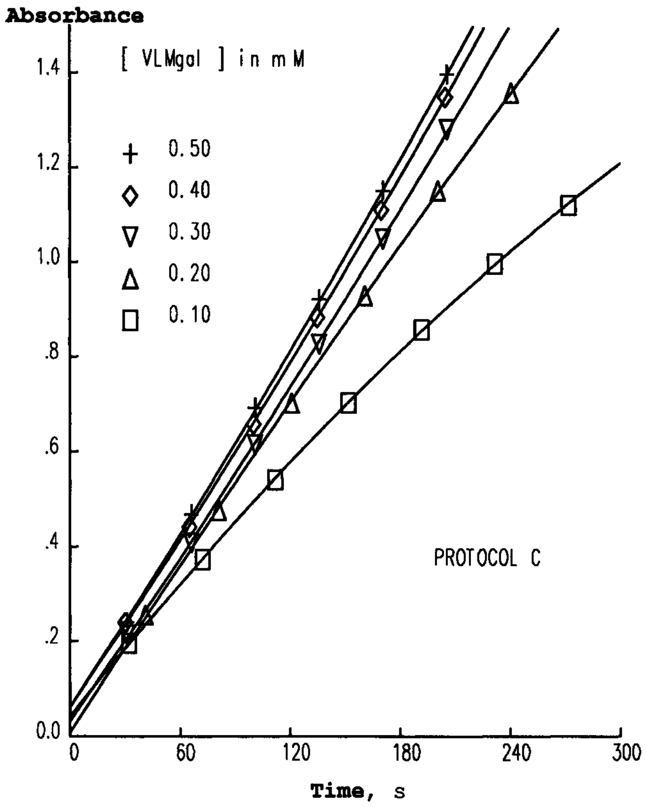

*Figure 3. Flow injection peak heights obtained for VLMgal-β-galactosidase using Protocol C.*

*Table 4. Double-reciprocal data for the Lineweaver-Burk plot.*

| Protocol A | | Protocol B | | Protocol C | |
|---|---|---|---|---|---|
| 1/[S], $mM^{-1}$ | $1/v_i$, $min^{-1}$ | 1/[S], $mM^{-1}$ | $1/v_i$, $min^{-1}$ | 1/[S], $mM^{-1}$ | $1/v_i$, $min^{-1}$ |
| 4·00 | 4·12 | 4·00 | 14·33 | 2·00 | 2·80 |
| 5·00 | 4·82 | 5·00 | 20·34 | 2·50 | 2·90 |
| 6·67 | 5·80 | 6·67 | 28·51 | 3·33 | 2·95 |
| 10·00 | 7·28 | 10·00 | 35·11 | 10·00 | 3·47 |
| 20·00 | 10·85 | | | | |

profiles as absorbance grows with time. To assess the performance of each protocol, the Michaelis constant was calculated and compared. The initial rates of individual assays were estimated by measuring the gradient during the first few moments of the reaction. The data for the double-reciprocal plots are presented in table 4 and plotted in figure 4.

The Lineweaver-Burk plots possess the following characteristics and the calculated values for $K_M$ and $V_{max}$ are also shown in table 5.

### Discussion

Mottola [9] has already demonstrated the advantage of using GOD in solution for the determination of glucose. It is therefore surprising that workers in the FIA field have

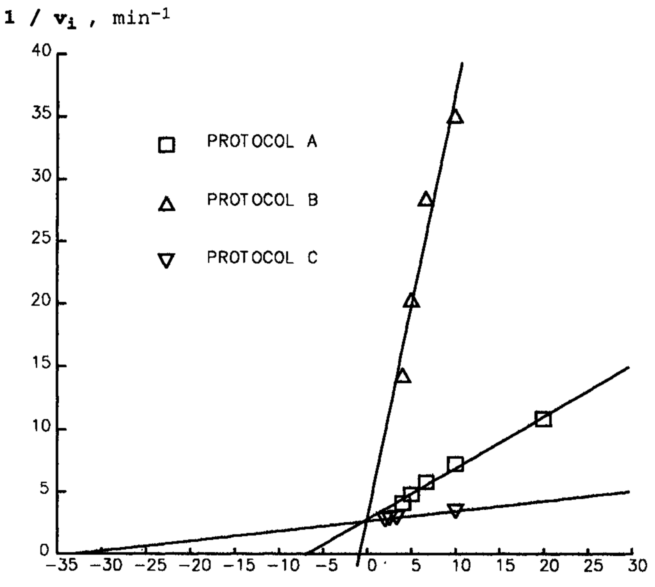

*Figure 4. Double reciprocal plots for VLMgal-β-galactosidase using the three protocols studied.*

*Table 5. Calculated $K_M$ and $V_{max}$ values for the three protocols.*

| | Protocol A | Protocol B | Protocol C |
|---|---|---|---|
| Slope | 0·4089 | 3·3495 | 0·0799 |
| Y-intercept | 2·8392 | 3·0794 | 2·6744 |
| X-intercept | −6·94 | −0·92 | −33·47 |
| $K_M$, mM | 0·14 | 1·09 | 0·03 |
| $V_{max}$ | 0·35 | 0·33 | 0·37 |

been reluctant to use the enzyme in this form. Reagent consumption could be a drawback in conventional peristaltic pump-based FIA systems, since the reagents would have to be pumped continuously in order to maintain a steady flow within the manifold. Attempts to reduce reagent consumption by pump-switching could disturb the flow of the system and lower the reproducibility. As previously described, the computer-controlled valve-switching and propulsion by gas-displacement has ensured that reagent consumption is minimal, whilst retaining maximum reproducibility. The colorimetric reaction developed by Barham and Trinder [4] was chosen because of its specificity to β-D-glucose and its freedom from potential interferents. Reducing agents, such as uric acid and creatinine, have no effect on the assay. Ascorbic acid only interferes in amounts far in excess of normal bodily levels. The glucose assay was adapted to the analyser only as a preliminary investigation and therefore no 'real' samples were analysed. Blood samples could be analysed by incorporating a dialyser unit along the manifold.

The protocols for the study of β-galactosidase–VLMgal interaction also revealed some interesting results. It was immediately apparent in comparing 'A' and 'B' that the presence of magnesium ions had enhanced the reaction for, although a much higher temperature was used in 'B', the absence of magnesium had resulted in a large reduction in the activity. Where magnesium was present in both protocols, the effect of temperature was greater than that of pH, as in the case of 'A' and 'C'. The maximal activity for cleavage of ONPG shifted from pH 6·8 for 2 mM $Mg^{++}$ to pH 7·4 for no magnesium at all [15]. This was almost a reversal of the case for ONPG, where the alkaline solution yielded the higher activity by virtue of its temperature. Another explanation may be the pH in which the chromophoric product was developed. It would probably be quicker for the solutions in 'C' to attain a pH of 10 when injected into the carrier than the solutions in 'A'. Substrate inhibition was most easily observed in 'C' where the kinetic profiles converge with the increase in substrate concentrations. The $K_M$ and $V_{max}$ values were 0·12 mM and 0·52 respectively when estimated manually and using a protocol containing 3 mM $Mg^{++}$, pH 7·3 and 37 °C [17]. The Michaelis constant obtained in protocol 'A' appeared to be very close to the value obtained manually. However, this finding should be viewed with caution, because the conditions for the reactions were not the same. The values obtained for the maximal activity, $V_{max}$, for the three protocols were very close to each other. These experiments were undertaken as a preliminary assessment of the suitability of VLMgal as a substrate for β-galactosidase in this type of experiment. The effect of alcohols on the cleavage of the substrate by the enzyme was not investigated.

### Conclusion

These results illustrate that the assay method is satisfactory, and the work is being extended to other enzymatic systems of interest in the hope of developing reliable FIA procedures for enzyme assays.

### References

1. Clark, J. M. Jr. and Switzer, R. L., *Experimental Biochemistry*, 2nd edn (W. H. Freeman and Company, San Francisco, 1977).
2. Holme, D. J. and Peck, H., *Analytical Biochemistry* (Longman, London, 1983).
3. Bergmeyer, H. U., *Methods of Enzymatic Analysis*, 2nd edn, Vol. 111 (Verlag Chemie, Weinheim, 1974).
4. Barham, D. and Trinder, P., *Analyst*, **97** (1972), 142.
5. Hansen, E. H., Ruzicka, J. and Rietz, B., *Analytica Chimica Acta*, **89** (1977), 241.
6. Worsfold, P. J., *Analytical Proceedings*, **22** (1985), 358.
7. Mottola, H. A., *Analyst*, **112** (1987), 719.
8. Hansen, E. H., *Analytica Chimica Acta*, **216** (1989), 257.
9. Mottola, H. A., *Analytica Chimica Acta*, **145** (1983), 27.
10. Roehrig, P., Wolff, C.-M. and Schwing, P., *Analytica Chimica Acta*, **153** (1983), 181.
11. Gorton, L. and Ogren, L., *Analytica Chimica Acta*, **130** (1981), 45.
12. Worsfold, P. J., *Analytica Chimica Acta*, **164** (1984), 103.
13. Olsson, B., Stalbom, B. and Johansson, G., *Analytica Chimica Acta*, **179** (1986), 203.
14. Stryer, L., Biochemistry, 2nd edn (W. H. Freeman and Company, San Francisco, 1981).
15. Wallenfels, K. and Weil, R., b-Galactosidase, pp. 618–663, in *The Enzymes*, Vol. III, Ed. Boyer, P. D. (Academic Press, London, 1972).

16. Aamlid, K. H., Lee, G., Smith, B. V. and Richardson, A. C., *Carbohydrate Research,* **C5** (1990), 205.
17. Aamlid, K. H., Lee, G., Smith, B. V., Richardson, A. C., Price, R. G. and Taylor, S. A., *Chemical Industry* (1989), 106.
18. Olsen, S., Ruzicka, J. and Hansen, E. H., *Analytica Chimca Acta,* **136** (1982), 101.
19. Riley, C. *et al., Journal of Automatic Chemistry,* **5** (1983), 32.
20. Kroner, K. H. and Kula, M. R., *Analytica Chimica Acta,* **163** (1984), 3.
21. Holm, K. A., *Analytica Chimica Acta,* **188** (1986), 285.
22. Holm, K. A., *Analyst,* **111** (1986), 927.
23. Yoza, N. *et al., Chemical Letters* (1983), 1433.
24. Yoza, N. *et al., Journal of Chromatography,* **325** (1985), 385.
25. Keller, J. W., *Analytical Letters,* **17(B7)** (1984), 589.
26. Malcolme-Lawes, D. J., Pasquini, C. and Wong, K. H., *Laboratory Microcomputer,* **8** (1989), 44.
27. Malcolme-Lawes, D. J. and Wong, K. H., *Analyst,* **115** (1990), 65.

